# Biological characterisation of the emerged highly pathogenic avian influenza (HPAI) A(H7N9) viruses in humans, in mainland China, 2016 to 2017

**DOI:** 10.2807/1560-7917.ES.2017.22.19.30533

**Published:** 2017-05-11

**Authors:** Wenfei Zhu, Jianfang Zhou, Zi Li, Lei Yang, Xiyan Li, Weijuan Huang, Sumei Zou, Wenbing Chen, Hejiang Wei, Jing Tang, Liqi Liu, Jie Dong, Dayan Wang, Yuelong Shu

**Affiliations:** 1 National Institute for Viral Disease Control and Prevention, China Centers for Disease Control and Prevention, Key Laboratory for Medical Virology, National Health and Family Planning Commission, Beijing, China; 2 These authors contributed equally to this work

**Keywords:** Highly pathogenic H7N9 viruses, HPAI, Receptor binding profile, Antigenic analysis, Drug sensitivity

## Abstract

With no or low virulence in poultry, avian influenza A(H7N9) virus has caused severe infections in humans. In the current fifth epidemic wave, a highly pathogenic avian influenza (HPAI) H7N9 virus emerged. The insertion of four amino acids (KRTA) at the haemagglutinin (HA) cleavage site enabled trypsin-independent infectivity of this virus. Although maintaining dual receptor-binding preference, its HA antigenicity was distinct from low-pathogenic avian influenza A(H7N9). The neuraminidase substitution R292K conferred a multidrug resistance phenotype.

Five outbreak waves have occurred since the low-pathogenic avian influenza A(H7N9) virus (LPAI H7N9) first emerged in spring 2013 in eastern China [1]. Highly pathogenic avian influenza A(H7N9) (HPAI H7N9) viruses, derived from their LPAI H7N9 counterparts, have recently been isolated from humans and resulted in fatal outcome in Guangdong, China (A/Guangdong/17SF003/2016 (SF003) and A/Guangdong/17SF006/2017 (SF006)) [2]. Both viruses contain an insertion of four amino acids (KRTA) in the haemagglutinin (HA) proteolytic cleavage site, indicating their pathotype switch from LPAI to HPAI. Furthermore, they retain a series of genetic features contributing to the ability to infect humans (e.g. 186V in the HA protein (H3 numbering) and 627K in the PB2 protein) that raise concerns regarding their pandemic potential. Amino acid substitutions associated with resistance to neuraminidase inhibitors (NAIs) have been detected in both SF003 and SF006 viruses. Therefore, to update public health risk assessment, we investigated trypsin-dependent infectivity, receptor binding properties, antigenic alternations of the HPAI H7N9 viruses, as well as their sensitivity to antiviral drugs. All LPAI H7N9 viruses in this study were isolated from humans.

## Similar replication ability of HPAI H7N9 viruses with or without trypsin

**Figure 1:**
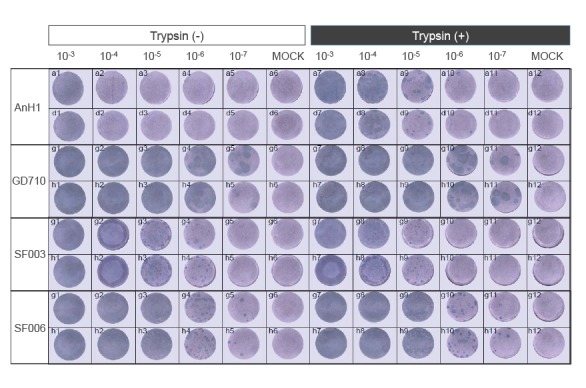
Replication of highly pathogenic avian influenza A(H7N9) viruses from humans in MDCK cells with or without TPCK trypsin, China, 2016­–2017 (n = 2)

AnH1: A/Anhui/1/2013 (LPAI H7N9); GD710: A/Guangdong/99710/2014 (HPAI H5N6); MDCK: Madin-Darby canine kidney epithelium; PBS: phosphate-buffered saline; SF003: A/Guangdong/17SF003/2016 (HPAI H7N9); SF006: A/Guangdong/17SF006/2017 (HPAI H7N9); TPCK: N-p-tosyl-L-phenylalanine chloromethyl ketone-treated.

Avian influenza viruses with multiple basic amino acids at the HA cleavage site are able to replicate in cell culture in the absence of N-p-tosyl-L-phenylalanine chloromethyl ketone-treated (TPCK) trypsin [3]. We therefore examined the in vitro growth of SF003 and SF006 in MDCK cells in the presence or absence of TPCK trypsin. Cells were fixed with ice-cold 4% paraformaldehyde complemented with Triton X-100 and detected by staining of the nucleoprotein (NP). LPAI H7N9 virus (A/Anhui/1/2013 (AnH1)) and HPAI H5N6 virus (A/Guangdong/99710/2014 (GD710)) were included as control. The three HPAI viruses (GD710, SF003 and SF006) had comparable ability to replicate both in the presence and absence of TPCK trypsin, while the LPAI H7N9 AnH1 virus failed to replicate in the absence of TPCK trypsin (Figure 1).

Serial viral dilutions of 10^−3^, 10^−4^, 10^−5^, 10^−6^ and 10^−7^ TCID_50_ were inoculated on MDCK cells. Mock cells were inoculated with PBS.

## Dual receptor-binding profile of HPAI H7N9 viruses

**Figure 2:**
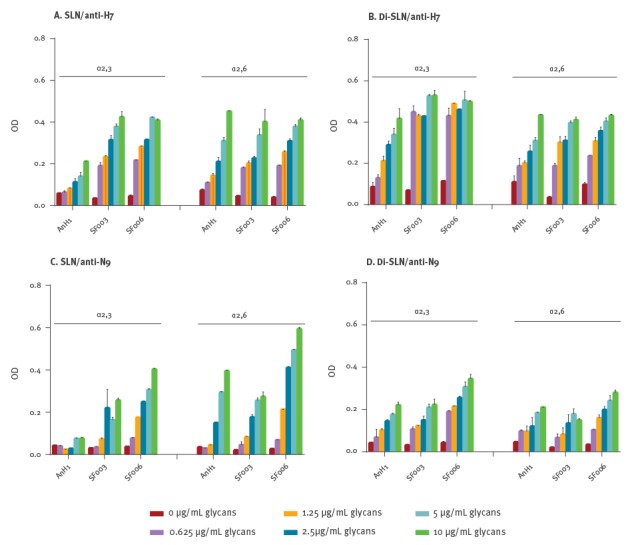
Direct glycan receptor binding of highly pathogenic avian influenza A(H7N9) viruses from humans, China, 2016–2017 (n =2)

AnH1: A/Anhui/1/2013 (LPAI H7N9); LN: lactosamine (Galβ1–4GlcNAc); OD: optical density; SF003: A/Guangdong/17SF003/2016 (HPAI H7N9); SF006: A/Guangdong/17SF006/2017 (HPAI H7N9); 3’SLN: Neu5Acα2–3 linked to LN; 6’SLN: Neu5Acα2–6 linked to LN.

HA amino acids 226L/I and 186V have been reported to contribute to the dual receptor binding properties of the LPAI H7N9 viruses [4,5]. However, 226L has mutated back to 226Q in the HPAI H7N9 viruses. In order to verify whether the receptor-binding profile of HPAI H7N9 differed from the LPAI viruses, we conducted a binding assay with synthetic sialylglycopolymers. The sialylglycopolymers, including 3’-SLN, 3’-SLN-LN, 6’-SLN and 6’SLN-LN (LN corresponds to lactosamine (Galβ1–4GlcNAc) and 3’SLN and 6’SLN, respectively, correspond to Neu5Ac α2,3 and Neu5Ac α2,6 linked to LN), were obtained from the Consortium of Functional Glycomics (http://www.functionalglycomics.org). As described previously [6,7], 32 haemagglutinating units (HAU) of live virus per well were used in the ELISA test. Binding was detected by a human chimeric anti-H7 monoclonal antibody (MoAb) or mouse-derived anti-N9 MoAb. The optical density was read at 450/630 nm. As shown in Figure 2, the LPAI H7N9 AnH1 virus bound to sialic acid α2,3 and α2,6 receptors, as expected. The HPAI H7N9 viruses SF003 and SF006 showed typical dual receptor preference, with increased affinity to α2,3 receptors compared with the LPAI H7N9 AnH1 virus. When using the anti-N9 monoclonal antibody, SF006 virus showed a slightly enhanced binding preference for α2,6 receptors compared with the AnH1 virus (Figure 2C). This phenotype may be due to the binding of N9 to the human-type receptor as reported recently [8].

Each sample was tested in duplicate. Absorbance was read at 450 nm. Binding profiles of tested viruses are presented with glycans of 0, 0.625, 1.25, 2.5, 5 and 10 μg/mL.

## Divergent antigenic properties of HPAI relative to LPAI H7N9 viruses 

Compared with the sequences of LPAI H7N9 viruses, several substitutions, including I38T, S112P, K164E and I317V (H7 numbering) have occurred in the HA1 protein of HPAI H7N9 viruses. To investigate the antigenic difference among HPAI and LPAI H7N9 viruses, a haemagglutination inhibition (HI) assay was conducted using the ferret anti-sera against A/Anhui/1/2013 (wildtype), A/Anhui/1/2013 (reverse genetics) and A/Shanghai/2/2013 (wildtype) according to standard protocols [9] (Table 1). Antigenic analysis demonstrated that all LPAI H7N9 viruses except A/Hunan/06948/2017 and A/Anhui/60933/2016 reacted well with ferret antiserum of H7N9 vaccine strains AnH1 (either wild-type or reverse genetics strains) or A/Shanghai/2/2013 (Table 1). However, both HPAI H7N9 viruses (SF003 and SF006) showed low or no reactivity to antisera of LPAI H7N9 vaccine strains.

**Table 1:** Haemagglutination inhibition reactions of human influenza A(H7N9) virus isolates, China, 2016–2017 (n = 16)

Strains	HA	Titre of ferret antiserum to antigen		
		AnH1	AnH1-RG	SH2
A/Anhui/1/2013	256	**160**	80	320
A/Anhui/1/2013-RG	256	320	**160**	640
A/Shanghai/2/2013	128	320	160	**640**
A/Shanghai/2/2013-RG	64	320	160	640
A/Fujian/02152/2017	1,024	160	40	160
A/Anhui/60936/2016	1,024	80	40	80
A/Jiangsu/06463/2017	1,024	320	80	160
A/Jiangsu/06454/2017	128	80	40	160
A/Jiangsu/60466/2016	32	160	40	80
A/Fujian/54840/2016	2,048	160	40	160
A/Jiangsu/60460/2016	2,048	80	40	80
A/Hunan/02287/2017	512	160	40	80
A/Guangdong/60060/2016	512	320	160	640
A/Guangdong/17SF004/2017	2,048	320	160	640
A/Guangdong/60923/2016	32	320	80	320
A/Guangdong/60061/2016	2,048	160	80	320
A/Hunan/06948/2017	2,048	40	< 40	80
A/Anhui/60933/2016	32	< 40	< 40	< 40
A/Guangdong/17SF003/2016	256	< 40	< 40	< 40
A/Guangdong/17SF006/2017	512	40	< 40	40

AnH1: A/Anhui/1/2013; HA: haemagglutinin; RG: reverse genetic; SH2: A/Shanghai/2/2013.

Homologous titres are indicated in bold.

## Multiple drug resistance of HPAI H7N9 viruses containing the R292K substitution

The substitution R292K (N2 numbering), which is associated with reduced susceptibility to NAIs [4], has been reported in the HPAI H7N9 viruses. Considering the possible effect that quasispecies containing NA-292R/K may have on drug susceptibility, we first purified SF003 virus from plaques. Two virus clones with either amino acid 292K or 292R in the NA protein, were analysed in a neuraminidase inhibition assay [10]. The susceptibility of the viruses was categorised by the criteria recommended by the World Health Organization (WHO) Antiviral Working Group [11]. As expected, the influenza A(H3N2) wild-type virus (A/Beijing-Haidian/1942/2014) and the LPAI H7N9 wild-type virus (AnH1) were sensitive to the three NAIs, while the A/Texas/12/2007 virus which contained the E119V substitution in the NA protein showed highly reduced inhibition to oseltamivir (mean: 798-fold increase in IC_50_) but normal inhibition to zanamivir and peramivir (Table 2). The HPAI H7N9 virus SF003 with NA-292R was similar to AnH1 and sensitive to all the three NAIs. However, the substitution R292K in the NA protein induced a mean 53,855-fold increase in the IC_50_ of oseltamivir, and a 3,556-fold and 73-fold increase in peramivir and zanamivir, respectively.

**Table 2:** Susceptibility of human isolates of highly pathogenic avian influenza A(H7N9) viruses to neuraminidase inhibitors, China, 2016 (n =2)

Viruses	Oseltamivir		Zanamivir		Peramivir	
	Mean IC_50_ (nM) ± SD	Fold change^a^	Mean IC_50_ (nM) ± SD	Fold change^b^	Mean IC_50_ (nM) ± SD	Fold change^a^
A/Anhui/1/2014 (H7N9)	0.58 ± 1.03	1.0	0.66 ± 1.07	1.0	0.05 ± 1.02	1.0
A/Guangdong/17SF003/2016 (H7N9–292R) ^b^	0.84 ± 1.06	1.4	0.95 ± 1.12	1.4	0.07 ± 1.04	1.4
A/Guangdong/17SF003/2016 (H7N9–292K) ^b^	31236.00 ± 1.68	53,855.2	69.33 ± 1.10	73.0	248.90 ± 1.18	3,555.7
A/Beijing-Haidian/1942/2014 (H3N2)	0.12 ± 1.14	1.0	0.12 ± 1.09	1.0	0.06 ± 1.04	1.0
A/Texas/12/2007 (H3N2-E119V) ^b^	95.70 ± 1.07	797.5	0.93 ± 1.10	7.75	0.10 ± 1.06	1.7

HPAI: highly pathogenic avian influenza; IC_50_: 50% inhibitory concentration; SD: standard deviation.

^a^ Compared with that of wild-type viruses. A/Texas/12/2007 (H3N2-E119V) and A/Anhui/1/2014 (H7N9) were the wild-type influenza A(H3N2) and influenza A(H7N9) viruses, respectively.

^b^ N2 numbering.

## Discussion

Compared with other avian influenza viruses, LPAI H7N9 and HPAI H5N1 are of most concern because of their high mortality and morbidity. LPAI H7N9 poses a higher risk for humans than HPAI H5N1 because LPAI H7N9 could bind sialic acid α2,6 human-type receptors while HPAI H5N1 could not. Our data show that the HPAI H7N9 viruses retained dual receptor binding properties, with slightly increased binding preference for both receptors compared with LPAI H7N9 (AnH1) viruses. It is well known that human upper respiratory tissues and trachea contain mainly α2,6 receptors while lung tissue possesses mixtures of avian type α2,3 and human type α2,6 receptors [7,12]. The persisting preference for both avian- and human-type receptors of HPAI H7N9 viruses may result in their circulation in poultry and possible transmission among humans.

Vaccination is the primary measure to control the spread of influenza virus infection in humans. Previously, WHO recommended A/Anhui/1/2013 (LPAI H7N9) as the vaccine strain for influenza A(H7N9) virus. However, our data show that the newly emerged HPAI H7N9 viruses did not react strongly with the ferret antisera of LPAI H7N9 viruses. Therefore, WHO has recently recommended SF003 as an additional candidate vaccine virus.

Among the four commercially available NAIs, oseltamivir and zanamivir are the predominant NAIs for influenza prophylaxis and treatment [13]. Our results show that HPAI H7N9 viruses with the 292K amino acid in the NA protein exhibited multi-drug resistance. The viral neuraminidase could acquire the R292K substitution as early as 2 days after administration of the antiviral drug. Further assessment of the fitness of drug-resistant viruses is urgently needed.

In summary, although the highly pathogenic influenza A(H7N9) virus was thought to cause higher risk in poultry than the low-pathogenic virus, our study, especially regarding the receptor profile of HPAI H7N9 viruses, has implications on surveillance and control strategies not only in the animal sector but also for public health. Our study also highlighted the critical role of antiviral surveillance monitoring in the clinical management of influenza virus infection as an essential component of pandemic preparedness.
